# Pb-induced cellular defense system in the root meristematic cells of *Allium sativum *L

**DOI:** 10.1186/1471-2229-10-40

**Published:** 2010-03-02

**Authors:** Wusheng Jiang, Donghua Liu

**Affiliations:** 1 Library of Tianjin Normal University, Tianjin 300387, PR China; 2 Department of Biology, Tianjin Normal University, Tianjin 300387, PR China

## Abstract

**Background:**

Electron microscopy (EM) techniques enable identification of the main accumulations of lead (Pb) in cells and cellular organelles and observations of changes in cell ultrastructure. Although there is extensive literature relating to studies on the influence of heavy metals on plants, Pb tolerance strategies of plants have not yet been fully explained. *Allium sativum *L. is a potential plant for absorption and accumulation of heavy metals. In previous investigations the effects of different concentrations (10^-5 ^to 10^-3 ^M) of Pb were investigated in *A. sativum*, indicating a significant inhibitory effect on shoot and root growth at 10^-3 ^to 10^-4 ^M Pb. In the present study, we used EM and cytochemistry to investigate ultrastructural alterations, identify the synthesis and distribution of cysteine-rich proteins induced by Pb and explain the possible mechanisms of the Pb-induced cellular defense system in *A. sativum*.

**Results:**

After 1 h of Pb treatment, dictyosomes were accompanied by numerous vesicles within cytoplasm. The endoplasm reticulum (ER) with swollen cisternae was arranged along the cell wall after 2 h. Some flattened cisternae were broken up into small closed vesicles and the nuclear envelope was generally more dilated after 4 h. During 24-36 h, phenomena appeared such as high vacuolization of cytoplasm and electron-dense granules in cell walls, vacuoles, cytoplasm and mitochondrial membranes. Other changes included mitochondrial swelling and loss of cristae, and vacuolization of ER and dictyosomes during 48-72 h. In the Pb-treatment groups, silver grains were observed in cell walls and in cytoplasm, suggesting the Gomori-Swift reaction can indirectly evaluate the Pb effects on plant cells.

**Conclusions:**

Cell walls can immobilize some Pb ions. Cysteine-rich proteins in cell walls were confirmed by the Gomori-Swift reaction. The morphological alterations in plasma membrane, dictyosomes and ER reflect the features of detoxification and tolerance under Pb stress. Vacuoles are ultimately one of main storage sites of Pb. Root meristematic cells of *A. sativum *exposed to lower Pb have a rapid and effective defense system, but with the increased level of Pb in the cytosol, cells were seriously injured.

## Background

Lead (Pb) exists in many forms in natural sources throughout the world. According to the USA Environmental Protection Agency, Pb is one of the most common heavy metal contaminants in aquatic and terrestrial ecosystems and can have adverse effects on growth and metabolism of plants due to direct release into the atmosphere [1]. There have been many reports of Pb toxicity in plants [2], including disturbance and toxicity of mitosis and nucleoli [3,4], inhibition of root and shoot growth [5], induction of leaf chlorosis [6], reduction in photosynthesis [7] and inhibition and activation of enzymatic activities [5,8,9].

It is well known that the roots are the main route through which Pb enters plants [10], and about 90% of Pb is accumulated in roots of some plants [11]. Most Pb in roots is localized in the insoluble fraction of cell walls and nuclei, which is connected with the detoxification mechanism of Pb [10]. With increasing Pb concentration in cells, a series of alterations at ultrastructural level appear. Electron microscopy (EM) techniques are very useful in localizing Pb in plant tissues [12-14]. They make it possible to identify the main accumulations of Pb in cells and cellular organelles and observe alterations in cell ultrastructure [14-17]. Plants have a range of potential mechanisms at different levels that might be involved in the detoxification and thus tolerance to heavy metal stress [18]. The main detoxifying strategy of plants contaminated by heavy metals is the production of phytochelatins (PCs) [19]. PCs, a family of metal-induced peptides, are produced in plants upon exposure to excess heavy metals, such as Cu, Cd or Zn [18], and can be detected in plant tissues and cell cultures [20]. Several studies have reported that PCs can form complexes with Pb, Ag and Hg in vitro [21].

Although there is extensive literature relating to cellular levels and physiological studies on the influence of heavy metals on plants, Pb tolerance strategies of plants have not been fully explained yet [5,15,17]. *Allium sativum *L. is a potential plant for absorption and accumulation of heavy metals [22,23]. In a previous investigation, the effects of different concentrations (10^-5^, 10^-4 ^and 10^-3 ^M) of Pb on growth for 20 d were investigated in hydroponically grown *A. sativum*. Pb had significant inhibitory effects on shoot growth at high concentrations (10^-3 ^M), on roots at 10^-3 ^and 10^-4 ^M during the entire experiment [5]. In the present study, we used EM and cytochemistry to investigate ultrastructural alterations, i.e. in plasma membrane, dictyosomes, endoplasm reticulum (ER) and mitochondria, to identify the synthesis and distribution of cysteine-rich proteins induced by Pb and to explain the possible mechanisms of the Pb-induced cellular defense system in the root meristematic cells of *A. sativum*.

## Results

### Effect of Pb on subcellular structures of root-tip meristems

**Figure 1:**
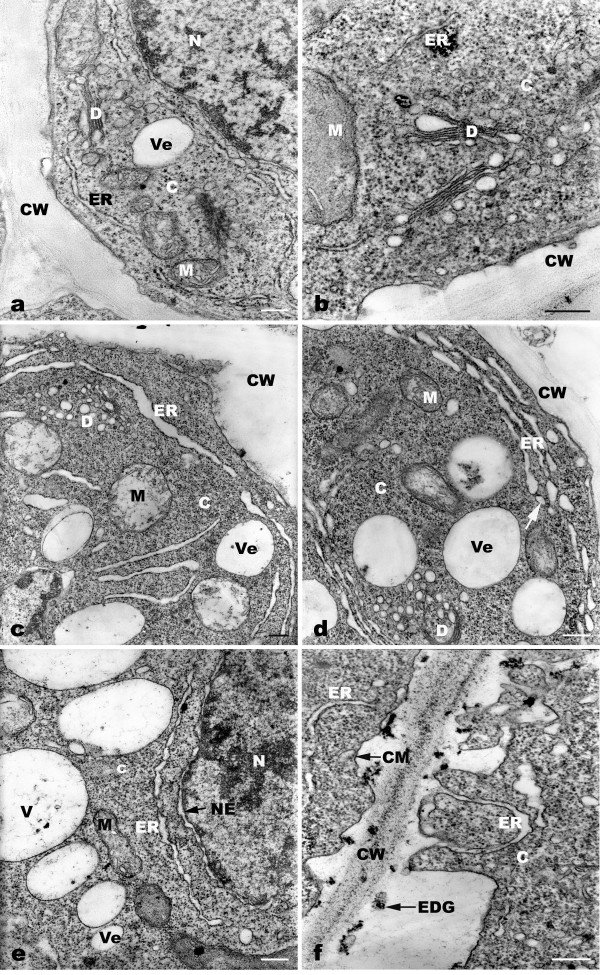
**TEM micrographs showing toxic effects of Pb on ultrastructure of the root meristematic cells of *A. sativum***. a: Control cells showing well-developed root tip cells. b-f: The ultrastructural changes of root meristematic cells exposed to 10^-4 ^M Pb for 1-2 h. b: Obvious increase in dictysome vesicles and formation of increasing numbers of vesicles near the cell wall at exposure for 1 h. c: Large amount of ER near the cell wall and some with distinct dilation of flattened cisterna after Pb treatment for 2 h. d: Flattened cisternae broken up into small closed vesicles (arrow). e: The nuclear envelope swelling in the root meristem after treatment for 4 h. f: Cytoplasm membrane invaginations (arrow) and active phagocytosis during the 4-h treatment. C = cytoplasm, CM = cytoplasm membrane, CW = cell wall, D = dictyosome, ER = endoplasmic reticulum, EDG = electron-dense granules, M = mitochondria, N = nucleus, NE = nuclear envelope, V = vacuole, Ve = vesicle. Bar = 0.25 μm.

Ultrastructural studies of the root tip cells of *A. sativum *grown in control solution and in solutions containing 10^-4 ^M Pb for different durations of time revealed extensive differences. Control cells had typical ultrastructure. Plasma membrane was unfolded with a uniform shape in all parts. Large amounts of rough ER, dictyosomes, mitochondria and ribosomes were immersed in dense cytoplasm. The nuclei with well-stained nucleoplasm and distinct nucleolus were located in the center of cells, whereas vesicles were distributed in root tip cells (Figure 1a).

**Figure 2:**
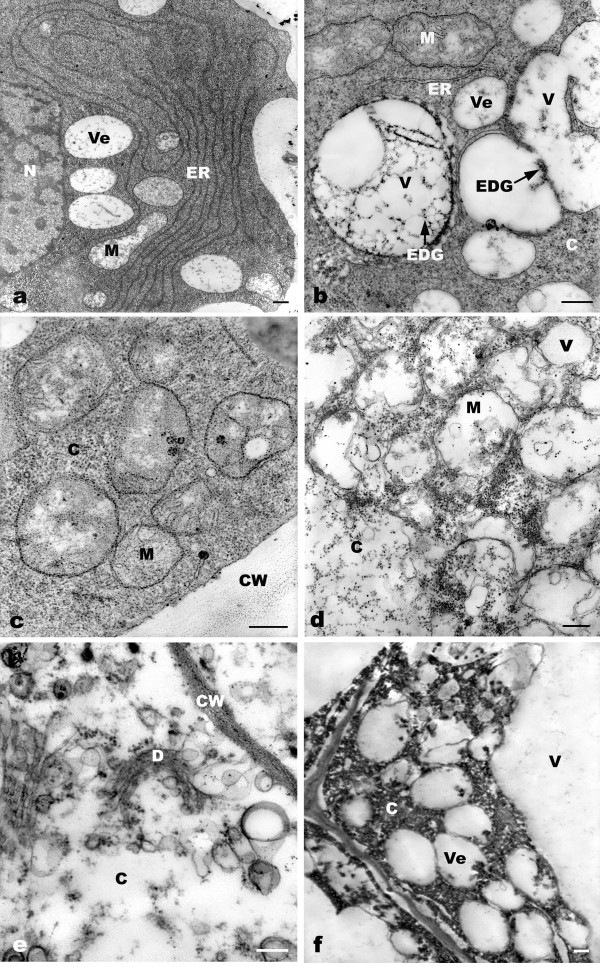
**TEM micrographs showing toxic effects of 10^-4 ^M Pb on ultrastructure of the root meristematic cells of *A. sativum***. a: Rich parallel arrays of ER with regularly extended cisternae (24 h). b: Increased vesicles from dictyosomes and ER, with some incorporated into bigger vacuoles; and accumulation of electron-dense granules containing Pb ions in vacuoles (arrows). c: Electron-dense granules localized on the surface of membranes in mitochondria. d: Obvious decrease in mitochondrial cristae and vesiculation of dictyosomes and ER (48 h). e. Vacuolization of dictyosomes (72 h). f. Plasmolysis and some electron-dense granules from vesicles relocated into cytoplasm due to loss of vesicle membrane function (72 h). C = cytoplasm, CM = cytoplasm membrane, CW = cell wall, D = dictyosome, ER = endoplasmic reticulum, EDG = electron-dense granules, M = mitochondria, N = nucleus, V = vacuole, Ve = vesicle. Bar = 0.25 μm

After 1 h of treatment, the observable effect of Pb at ultrastructural level was that the dictyosome vesicles increased, appearing as a compact mass of vesicles in the cytoplasm (Figure 1b). After 2 h of Pb treatment, the ER with swollen cisternae appeared to be concentrically arranged along the cell wall (Figure 1c, d). Some flattened cisternae were broken up into small closed vesicles (Figure 1d). After treatment with Pb for 4 h, in some meristematic cells the nuclear envelope was generally more dilated compared with control cells (Figure 1e). There were marked invaginations of plasmalemma (Figure 1f). There were some small vesicles, containing electron-dense granules, formed by the plasma membrane. The morphological alterations above took place during 12 h of treatment with Pb, but no visible injury in other cellular components was seen. An interesting phenomenon was found at 24 h of Pb exposure; many parallel arrays of ER with regularly extended cisternae were noticeable in cytoplasm (Figure 2a). After 36 h of Pb treatment, there was high cytoplasmic vacuolization in root tip cells. Normally, several vesicles gradually fuse together to produce a large cytoplasmic vacuole, in which electron-dense granules can be seen (Figure 2b). The electron-dense granules were firstly found in cell walls and also deposited in spaces between the cell walls and plasma membrane (Figure 1f). Then there was a gradual accumulation of electron-dense granules in vacuoles, cytoplasm and mitochondrial membranes with increasing Pb treatment time (Figure 2c). Ultrastructural and morphological damage was observed during long exposure (48-72 h), revealing mitochondrial swelling, loss of cristae (Figure 2d), vacuolization of ER and dictyosomes (Figure 2e). Plasmolysis occurred in some cells and some cells disintegrated (Figure 2f). The nuclei were a deep color and with no obvious margin of nucleoli, and plasma membranes were injured.

### Cytochemical test: Gomori-Swift reaction

**Figure 3:**
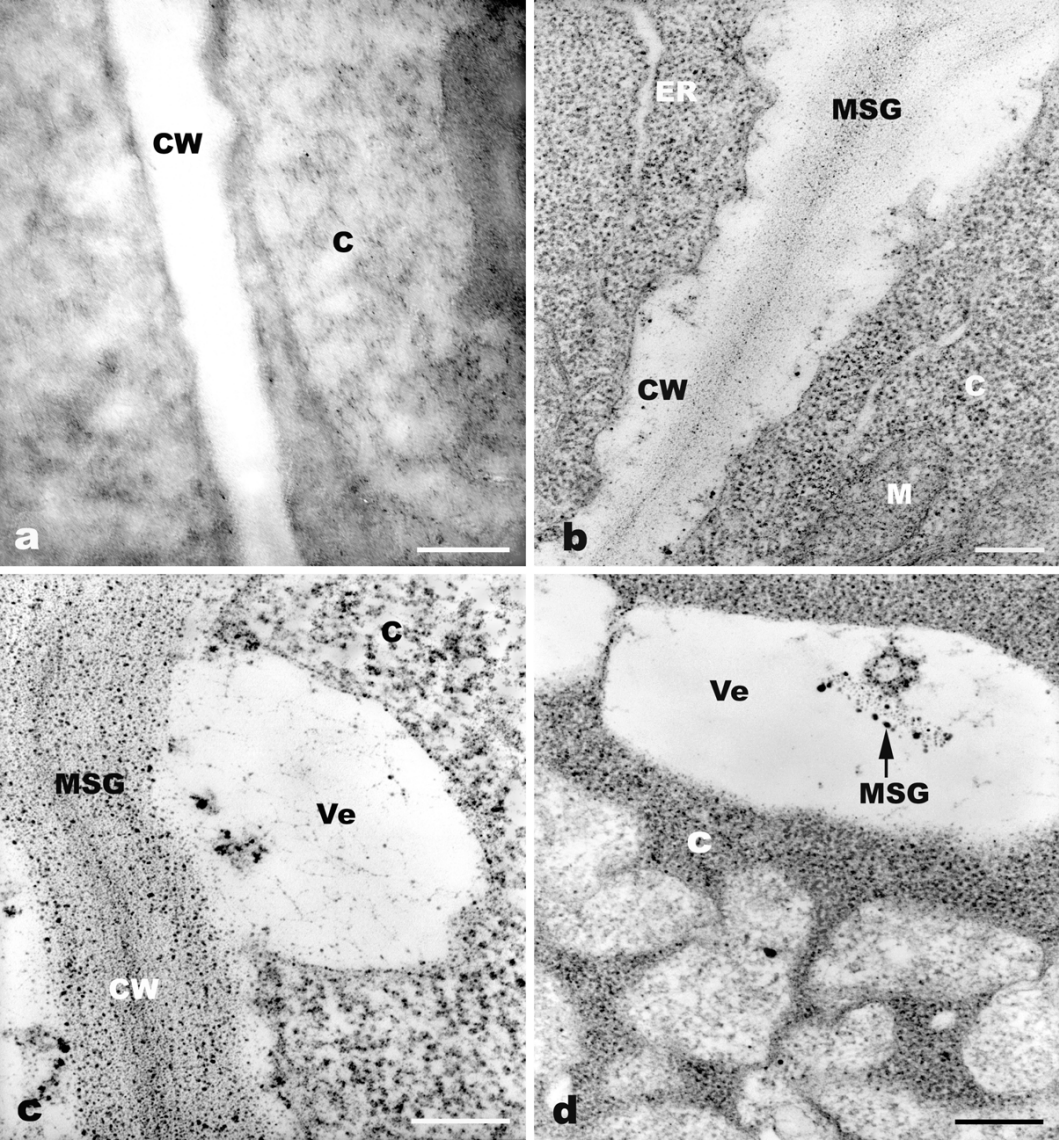
**TEM micrographs showing cytochemical test of the root meristematic cells of *A. sativum *exposed to 10^-4 ^M Pb**. a: No Gomori-Swift reaction in control cells. b: Trace amounts of silver grains in the cell walls of root cells exposed to Pb for 2 h. c: Increased amount of metallic silver grains in cell walls after treatment for 4 h. d: Rich metallic silver grains in cytoplasm and vesicles (arrow; 24 h). C = cytoplasm, CW = cell wall, ER = endoplasmic reticulum, M = mitochondria, MSG = metallic silver grains, Ve = vesicle. Bar = 0.25 μm.

The Gomori-Swift reaction is highly sensitive and allows the detection of cysteine-rich proteins in the cell. During the Gomori-Swift test treatment, silver nitrate and methenamine interact with cysteine from proteins. The hydroxyquinonold subunits of the melanin macromolecule can also reduce the silver-methenamine reagent. There were no metallic silver grains seen in the control root cells (Figure 3a). In the Pb treatment groups, three phenomena were noted. Firstly, trace amounts of silver grains were observed in the cell walls of meristematic cells after 2 h of exposure (Figure 3b). As a consequence of increased time of exposure to Pb from 4 h onward, they gradually increased in number (Figure 3c) and a large amount of silver grains accumulated for 24 h. Then, the Gomori-Swift reaction in cell walls gradually decreased with prolonged treatment time of Pb (72 h). Secondly, abundant metallic silver grains were distributed in cytoplasm (Figure 3b-d). Thirdly, small amounts of vesicles containing silver grains were distributed in cytoplasm (Figure 3d). Thus, the Gomori-Swift reaction can indirectly evaluate the toxic effects of Pb on plant cells under these conditions.

## Discussion

In previous work, the uptake and accumulation of Pb in *A. sativum *were investigated by inductively coupled plasma atomic emission spectrometry (ICP-AES), indicating that Pb accumulated primarily in roots; the concentration in bulbs and shoots was much lower [5]. When Pb enters cells, even in small amounts, it produces a wide range of adverse effects on physiological processes [9]. The ultrastructural results in the present investigation showed some electron-dense granules in vacuoles, cell walls and cytoplasm in the meristematic cells after Pb treatment. X-ray microanalysis of root cells of *Zea mays *[24] and *Allium cepa *[25] revealed that the electron-dense precipitates contained Pb ions. The increased amount of electron-dense granules in metal-exposed cells suggested that the formation of granules could be a detoxification pathway to prevent cell damage [26].

Our results here indicated that Pb ions were localized and accumulated in cell walls and vacuoles in *A. sativum*. Pb retention in the roots is based on binding of Pb to ion-exchange sites on the cell wall and extracellular precipitation, mainly in the form of Pb carbonate deposited in the cell wall [9]. Once excessive Pb ions enter the cytoplasm, a defense mechanism is activated, protecting the cells against Pb toxicity at the cellular level. Endocytotic and exocytotic processes are well known in plant cells. The plasma membrane represents a 'living' barrier of the cell to free inward diffusion of Pb ions. The results here indicated some vesicles containing Pb deposits were found in cells and were obviously derived from the invaginations of plasmalemma and ER. It was clearly shown that they could prevent the circulation of free Pb ions in the cytoplasm and could force them into a limited area. Mobilization and transport of metal ions across the plasma membrane are only the first steps in metal uptake and accumulation [27]. Plasma membrane function may be rapidly affected by heavy metals, as shown by increased leakage from cells in the presence of high concentrations of metals [18].

It is well known that the ER is the principal site of membrane synthesis within the cell. It appears to give rise to vacuolar and microbody membranes, as well as to the cisternae of dictyosomes in at least some plant cells [28]. Our results showed that root tip cells had a rapid and effective defense system against Pb toxicity involving ER and dictyosomes, which may be one mechanism accounting for lower toxicity of Pb. During 24 h of Pb exposure, the number of ER with regularly extended cisternae sharply increased (Figure 2a). This phenomenon may be explained by the fact that once excessive Pb ions entered cytoplasm, the synthesis of new proteins of ER involved in heavy metal tolerance was stimulated. We assume that some vesicles from ER and dictyosomes may carry metal-complexing proteins or polysaccharide components, which participated in repair of membrane and cell wall following damage. Some vesicles may have carried the proteins, which bind Pb by formation of stable metal-PC complexes in cytoplasm. In this way, the free metal ions in the cytoplasm decreased. Cells can maintain sufficient PCs to bind with Pb. ER definitely plays a very important role in detoxification of Pb.

The vacuole is the final destination for practically all toxic substances that plants can be exposed to, and the vacuoles of root cells are the major sites of metal sequestration [27]. Cytoplasmic vacuolization and the increased level of electron-dense granules in vacuoles can be thought of as a detoxification pathway for preventing cell damage and retaining the metal in specific vacuoles [26]. Sharma and Dubey indicated that within the cell the major part of Pb was sequestered in the vacuole in the form of complexes [9]. Pinocytosis is observed in leaf cells of many plants treated with Pb salt solutions. Through pinocytotic vesicles, Pb particles can be discharged into the vacuole [29].

Tolerance to metal stress relies on the plant's capacity to detoxify metals that have entered the cell. Inside cells, plant protection against metal toxicity involves synthesis of PCs and related peptides, organic acids and their derivatives [30]. Chelation of metals in the cytosol by high-affinity ligands is potentially a very important mechanism of heavy-metal detoxification and tolerance [18]. The PCs are cysteine-rich peptides that are enzymatically synthesized [19]. Estrella-Gomez suggested that the accumulation of PCs in *Salvinia minima *was a direct response to Pb accumulation, and PCs participate as one of the mechanisms to cope with Pb in this Pb-hyperaccumulator aquatic fern [31]. PC binds to Pb ions leading to sequestration of Pb ions in plants and thus serves as an important component of the detoxification mechanism in plants [9].

The histochemical test by Gomori-Swift reaction is highly sensitive and allows the detection of cysteine-rich proteins where toxic elements were usually detected [32]. Evidence from this cytochemical test confirms that cysteine-rich proteins, commonly referred to as PCs, were localized in cell walls and vesicles, and distributed in cytoplasm. The cysteine-rich proteins in cell walls were exhibited after roots were exposed to Pb solution for 2 h, indicating that Pb ions can induce synthesis of PCs. Skowroñski et al. [33] showed that in the green microalga *Stichococcus bacilaris*, PCs were detected after only 30 min of Cd exposure. In the presence of excess metals, PCs are formed and effectively capture metals [27]. Piechalak et al. demonstrated that the synthesis of thiol peptides could take place under the influence of Pb ions in root cells of three tested plant species of the Fabaceae family: *Pisum sativum, Vicia faba *and *Phaseolus vulgaris *[10]. They found that high amounts of these peptides were formed in the roots of *P. sativum*, despite the fact that this plant had a medium-tolerance index value, while the concentration of PCs in the roots of *V. faba *was much lower but their induction took place after only 2 h. The results showed that the rapid initiation of this cytoplasmic detoxification system, which consists of PCs, could transport Pb-PC complexes through the cytosol into vacuoles at lower concentrations of heavy metals [10]. Thus the PC pathway consists of two parts, metal-activated synthesis of peptides and transport of the metal-PC complexes into the vacuole [27].

## Conclusions

The results of the present and previous studies strongly suggest that: (1) cell walls, a first barrier against Pb stress, can immobilize some Pb ions. The cysteine-rich proteins in cell walls were confirmed by the Gomori-Swift reaction; (2) the morphological alterations in plasma membrane, dictyosomes and ER reflect the features of detoxification and tolerance under Pb stress; and (3) vacuoles are ultimately one of the main storage sites of Pb. Thus, root meristematic cells of *A. sativum *exposed to low Pb concentrations have a rapid and effective defense system, but at increased levels of Pb in the cytosol, cells are seriously injured.

## Methods

### Plant material and metal treatments

Healthy and equal-sized cloves of *Allium sativum *L. were chosen and allowed to form roots in containers of modified Hoagland's nutrient solution [34]. Plants were grown in a greenhouse equipped with a supplementary light with a 15/9-h light/dark diurnal cycle at 18-20°C. The Hoagland solution consisted of 5 mM Ca(NO_3_)_2_, 5 mM KNO_3_, 1 mM KH_2_PO_4_, 50 μM H_3_BO_3_, 1 mM MgSO_4_, 4.5 μM MnCl_2_, 3.8 μM ZnSO_4_, 0.3 μM CuSO_4_, 0.1 mM (NH_4_)_6_Mo_7_O_24 _and 10 μM FeEDTA at pH 5.5. Pb was provided as lead nitrate (Pb(NO_3_)_2_). The controls were grown on Hoagland solution alone. Seedlings were exposed to 10^-4 ^M Pb for 1, 2, 4, 8, 12, 24, 36, 48 and 72 h.

### Transmission electron microscopy

The terminal portion (about 2 mm) of each root of the control and the treated groups were cut and fixed in a mixture of 2% formaldehyde and 2.5% glutaraldehyde in 0.2 M phosphate buffer (pH 7.2) for 2 h and then thoroughly washed with the same buffer three times. This was followed by post-fixation with 2% osmium tetroxide in the same buffer for 2 h. They were dehydrated in an acetone series, and embedded in Spurr's ERL resin. For ultrastructural observations, ultrathin sections of 75-nm thickness were cut on an ultramicrotome (Leica EM UC6, Germany) with a diamond knife, and were mounted in copper grids with 300 square mesh. The sections were stained with 2% uranyl acetate for 50 min and lead citrate for 15 min. Observation and photography were accomplished by transmission electron microscopy (JEM-1230, Joel Ltd, Tokyo, Japan).

### Cytochemical tests

The Gomori-Swift test was used in the present investigation to detect whether cysteine-rich protein was induced under Pb stress.

Sections of 100-nm thickness from fixed material were cut and mounted on gold grids. The Gomori-Swift reaction was performed in the solution obtained by mixing two components just before staining. Solution A containing 5 mL of 5% silver nitrate and 100 mL of 3% hexamethylenetetramine, and solution B consisting of 10 mL of 1 × 44% boric acid and 100 mL of 1 × 9% borax were prepared. The final stain was obtained by mixing 25 mL of A, 5 mL of B and 25 mL of distilled water [35,36].

The grids were floated in the silver methenamine solution for 90 min at 45°C in the dark, and then washed four times for 2 min. The grids were then floated on 10% sodium thiosulfate solution for 1 h at room temperature to dissolve metallic silver and rinsed in deionized water four times for 2 min. The sections were continuously stained with uranyl acetate and lead citrate.

Controls were carried out to block SH and SS groups by the reduction of disulfide bonds in benzylmercaptan, followed by alkylation of SH groups in iodacetate boric acid. The procedures were described by Swift [35] and Liu and Kottke [36].

## Authors' contributions

WJ carried out the present investigation, participated in sample preparation and observation and drafted the manuscript. DL conceived the study, and participated in its design and coordination and revised the manuscript. All authors read and approved the final manuscript.
